# A Novel Technique for Augmenting Venous Outflow in the Superior Gluteal Artery Perforator (SGAP) Flap

**Published:** 2019-10-21

**Authors:** Katherine H. Carruthers, Ergun Kocak, Pankaj Tiwari, Shunsuke Yoshida

**Affiliations:** ^a^Division of Plastic Surgery, Department of Surgery, West Virginia University, Morgantown; ^b^Midwest Breast and Aesthetic Surgery, Gahanna, Ohio

**Keywords:** venous insufficiency, SGAP, autologous reconstruction, CTA, perforator flap

**Figure:**
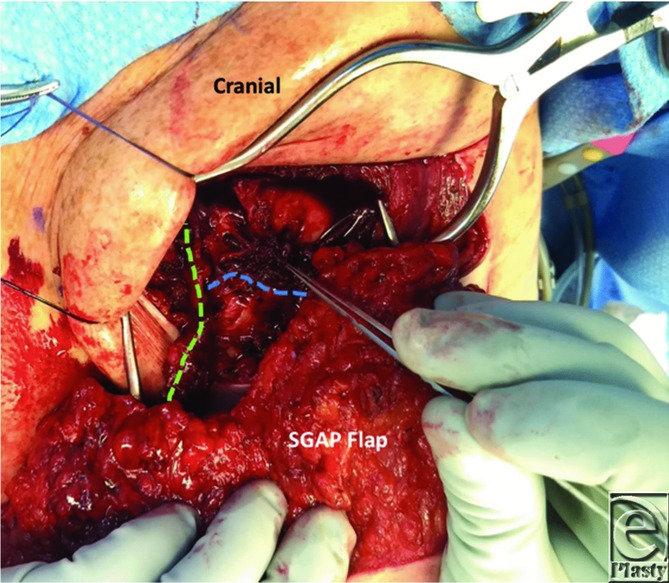
A Novel Technique for Augmenting Venous Outflow in the Superior Gluteal Artery Perforator (SGAP) Flap We herein describe a novel method for augmenting venous outflow in the SGAP flap in the setting of breast reconstruction.

In microvascular surgery, venous insufficiency is one of the most common complications leading to reoperation. To address this issue, techniques involving venous bypass or augmentation of venous outflow have been employed in a variety of free flap procedures. For example, the deep inferior epigastric artery perforator (DIEP) flap has a well-established method for augmenting the venous outflow by draining the superficial system.^1^^,^^2^ This technique has been shown to be an effective and reliable method for improving venous congestion.^2^

In recent years, there has been a growing trend to utilize alternative autologous tissue for breast reconstruction. The superior gluteal artery perforator (SGAP) flap, in particular, has become a common choice when DIEP flaps are not an option.^3^^-^5 The advantages of the SGAP flap are numerous, including the large volume of available tissue and the avoidance of an abdominal donor site, all while preserving the integrity of the gluteal muscle.^1^^,^^5^^,^^6^

While most surgeons agree that the SGAP flap is a technically demanding procedure due to anatomic variability during flap dissection and vessel mismatch at the anastomosis site, there are few reported techniques for SGAP flap salvage.^3^^,^^4^^,^^7^ Even in the most experienced of hands, there is still a risk of flap compromise, and if reoperation does occur, it is most often due to venous complications.^2^^,^^8^ Yet, to date, no prior studies have reported a reliable technique for augmenting venous flow in the SGAP flap. We herein report a method for augmenting venous outflow in the SGAP flap in the setting of autologous breast reconstruction.

## METHODS

### Surgical technique

**Figure 1:**
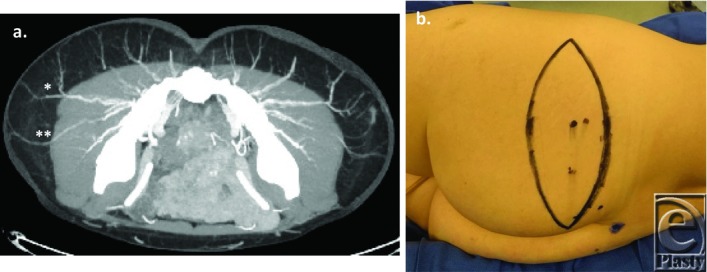
(*a*) CTA images were obtained at 2-mm intervals, along with 3-view thick slab maximum intensity projection images at 30-mm intervals. * indicates the dominant perforator. ** indicates the secondary, lateral perforator. (*b*) Vessel locations were confirmed and marked intraoperatively using a pencil Doppler probe. An elliptical-shaped skin island was designed so that the perforators were centrally placed.

Preoperatively, computed tomography angiography (CTA) imaging is obtained to identify the dominant and secondary perforators for the SGAP flap (Fig 1*a*).^5^^,^^9^ On the day of surgery, vessels that were identified on the CTA are confirmed intraoperatively using a pencil Doppler probe. A fusiform-shaped skin island is then designed to not only centralize the dominant perforator but also to include the secondary perforator (Fig 1*b*). The skin incisions are then made, and dissection is carried down to the gluteal fascia.^10^

**Figure 2:**
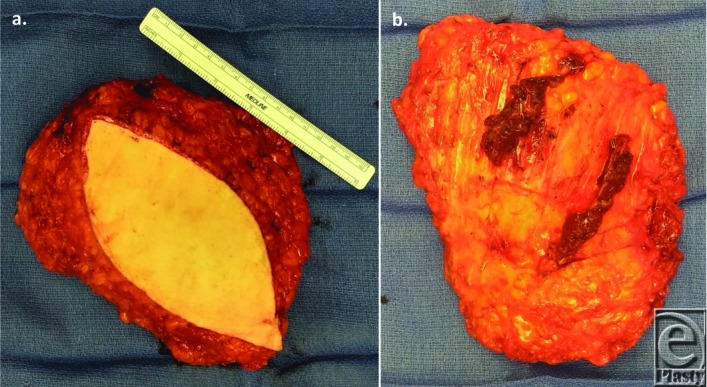
(*a*, *b*) During dissection, the 2 sets of perforators that were noted on the CTA were harvested. The flap was based on the central perforator, with the more lateral perforator preserved as a venous bypass should there be evidence of venous congestion.

Working from laterally to medially, the perforators are identified and carefully dissected while noting vessel length and caliber. Since the vessels are usually divided before they join their parent vessel, these perforators are essentially pedicles. Both pedicles consist of one artery and the accompanying veins (venae comitantes). We find that dissecting the structures as a unit facilitates the speed of the dissection and minimizes the risk of injury. For both perforators, approximately 6 cm of pedicle length is obtained before they are divided (Figs 2*a* and 2*b*).

**Figure 3:**
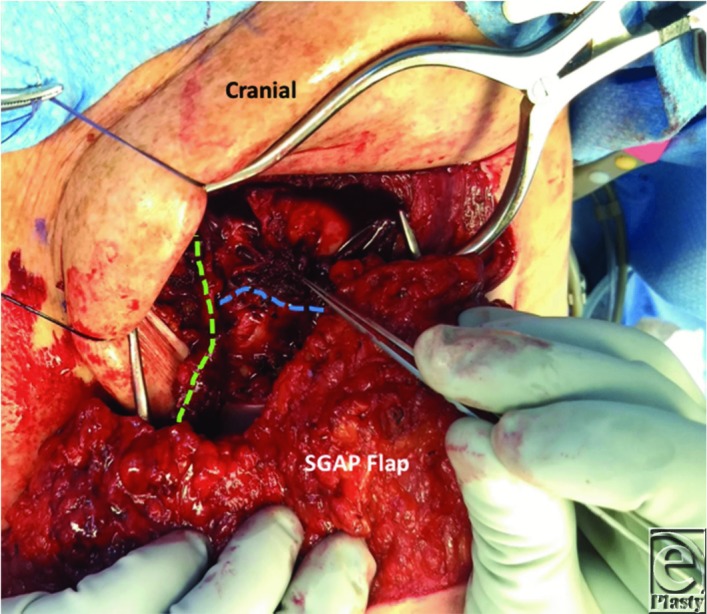
The flap was revascularized by anastomosing the vein of the dominant pedicle to the proximal IMV (green line) using a 2.0-mm coupling device and interrupted 8-0 nylon sutures for the arterial anastomosis. Clinical signs of venous congestion prompted the need for anastomosis of the second vein. This vessel was drained in a retrograde fashion into the distal IMV using a 2.0-mm venous coupling device (blue line). IMV indicates internal mammary vein.

The mastectomy incision is opened, and the recipient internal mammary vessels are dissected after resection of the third rib cartilage or through the intercostal space in a rib-sparing fashion. The flap is then revascularized by anastomosing the artery and vein of the centrally located perforator anterograde to the internal mammary vessels. At this time, clinical signs of venous congestion with a clearly patent venous anastomosis indicate venous insufficiency and prompt the need for anastomosis of the secondary perforator vein, which is anastomosed in a retrograde fashion to the distal internal mammary vein or in an anterograde fashion if an internal mammary perforator vein is present (Fig 3).

## RESULTS

In our initial experience using this technique to augment the venous outflow in the SGAP flap, we have found it to provide reliable and persistent improvement in venous congestion. While CTA guided the design of our flap skin island, we did not need to alter the position and dimensions of the flaps to include the second perforator, as they had a tendency to fall within the standard flap territory. Venous congestion was identified intraoperatively after successful anastomosis of the primary perforators of the superior gluteal artery and vein and subsequent anastomosis of an additional vena comitans resulted in clinically observable improvement in the venous congestion. It is important to note that there is often a dominant vena comitans in each pedicle, which contributes more to the overall venous outflow of the flap. Typically, one of the venae comitantes will be much larger than the other and, more importantly, quite attenuated. This vessel should be identified as dominate and used as the secondary venous outflow.

## DISCUSSION

Although the SGAP flap can be technically challenging, it serves as an important secondary donor site when other options such as thigh-based flaps are not ideal.^3^ Studies have shown that it is a robust flap that can tolerate a degree of vascular stress and has similar rates for hematoma, seroma, and reoperation when compared with DIEP flaps.^2^^,^^4^ There have been reports of bipedicled SGAP flaps for breast reconstruction described in the literature.^4^ However, in our experience, this angiosome is typically well perfused via a single arterial perforator and the addition of a supplementary, technically challenging, arterial anastomosis may not be needed in this setting. In contrast, preserving only an additional vein adds minimal time to the operation and allows for augmentation of venous outflow as a means for preventing SGAP flap venous congestion.

There is some degree of variability in the vascular anatomy, in terms of number, size, and location of perforators in the gluteal region, leading to an increased risk of vascular complication.^5^^,^^7^ Given these inherent challenges, the authors advocate for routine dissection of an additional vein that can be used as a lifeboat in cases of venous congestion. Although the exact venous arrangement described in this report may not always be present, similar minor perforating vessels are routinely noted on preoperative imaging and can easily be incorporated into the flap design.^8^

Venous congestion can occur despite a single patent venous anastomosis, and this can lead to flap failure if not recognized early and managed appropriately.^2^ We have proposed a technique for establishing reliable augmentation of venous outflow in SGAP flaps through anastomosis of a second vein. By using this method, one of the most common complications associated with autologous breast reconstruction can potentially be avoided, allowing the SGAP flap to more reliably be used for autologous breast reconstruction when other traditional flaps are not an option.

## SUMMARY AND IMPLICATIONS

We have proposed a technique for augmentation of venous outflow in SGAP flaps through anastomosis of a second perforating vein. By using this method, one of the most devastating complications associated with microsurgical reconstruction can potentially be avoided, allowing the SGAP flap to more reliably be used for breast reconstruction.
